# Getting more than they realized they needed: a qualitative study of women's experience of group prenatal care

**DOI:** 10.1186/1471-2393-12-17

**Published:** 2012-03-21

**Authors:** Deborah A McNeil, Monica Vekved, Siobhan M Dolan, Jodi Siever, Sarah Horn, Suzanne C Tough

**Affiliations:** 1 Public Health Innovation and Decision Support, Population and Public Health, Alberta Health Services, Southport Atrium, 10101 Southport Road SW, Calgary, Alberta T2W 3N2, Canada; 2 Faculty of Nursing, University of Calgary, 2500 University Drive NW, Calgary, Aberta T2N 1 N4, Canada; 3 Department of Paediatrics, Faculty of Medicine, University of Calgary, 2888 Shaganappi Trail NW, Calgary, Aberta T3B 6A8, Canada; 4 Department of Obstetrics & Gynecology and Women's Health, Albert Einstein College of Medicine/Montefiore Medical Center, Mazer 634, 1300 Morris Park Avenue, Bronx, NY 10461, USA; 5 Department of Community Health Sciences, Faculty of Medicine, University of Calgary, 3280 Hospital Drive NW, Calgary, Aberta T2N 4Z6, Canada

**Keywords:** Canada, Prenatal care, Pregnant women, Women's health, Social support

## Abstract

**Background:**

Pregnant women in Canada have traditionally received prenatal care individually from their physicians, with some women attending prenatal education classes. Group prenatal care is a departure from these practices providing a forum for women to experience medical care and child birth education simultaneously and in a group setting. Although other qualitative studies have described the experience of group prenatal care, this is the first which sought to understand the central meaning or core of the experience. The purpose of this study was to understand the central meaning of the experience of group prenatal care for women who participated in CenteringPregnancy through a maternity clinic in Calgary, Canada.

**Methods:**

The study used a phenomenological approach. Twelve women participated postpartum in a one-on-one interview and/or a group validation session between June 2009 and July 2010.

**Results:**

Six themes emerged: (1) "getting more in one place at one time"; (2) "feeling supported"; (3) "learning and gaining meaningful information"; (4) "not feeling alone in the experience"; (5) "connecting"; and (6) "actively participating and taking on ownership of care". These themes contributed to the core phenomenon of women "getting more than they realized they needed". The active sharing among those in the group allowed women to have both their known and subconscious needs met.

**Conclusions:**

Women's experience of group prenatal care reflected strong elements of social support in that women had different types of needs met and felt supported. The findings also broadened the understanding of some aspects of social support beyond current theories. In a contemporary North American society, the results of this study indicate that women gain from group prenatal care in terms of empowerment, efficiency, social support and education in ways not routinely available through individual care. This model of care could play a key role in addressing women's needs and improving health outcomes.

## Background

Pregnant women in Canada typically receive prenatal care through one-on-one visits with their physician or midwife. The Society of Obstetricians and Gynaecologists of Canada guidelines recommend that pregnant women have an initial prenatal visit with her provider within 12 weeks from the time of the her last menstrual period with subsequent visits increasing in later gestation [1]. Canada's universal health care system provides funding for prenatal visits with a physician, and most Canadian provinces provide funding for prenatal visits with a midwife [2]. In Alberta, the province where this study was conducted, the government began providing funding for midwifery services in 2009. In 2006, about 90% of pregnant women in Canada received prenatal care from a physician (58% from an obstetrician/gynaecologist and 34% from a family physician) with 6% receiving care from a midwife [3]. In the United Kingdom, Norway, Sweden, Finland, and New Zealand, there is a high proportion of midwives relative to each country's population, reflecting the fact that midwives deliver the majority of prenatal care in these countries [4-6]. This is in contrast with Canada and other countries, such as the United States and Germany, which have a low proportion of midwives relative to the country's population and where prenatal care is largely provided by physicians [4].

In addition to individual prenatal visits with their physician or midwife, some Canadian women also choose to attend prenatal classes. In prenatal classes, an educator provides pre-specified, educational material to a group of about 12 pregnant women and their partners or support people, and the material tends to focus on child birth. In Calgary, the city where this study was conducted, most prenatal classes start in the third trimester of pregnancy and vary in length from a total of 12 to 20 hours of instruction. Women are generally required to pay to attend most prenatal classes in Calgary.

Group prenatal care, an emerging alternative form of care, is gaining momentum. It is a departure from current practices for prenatal care and education, providing a forum for women to experience medical care and child birth education simultaneously and in a group setting. In CenteringPregnancy, a specific form of group prenatal care, pregnant women receive prenatal care over 10 two-hour sessions in a group of 8 to 12 women of similar gestational age [7]. Group sessions start early in the second trimester of pregnancy [7]. During each session, women participate in their own assessment by measuring and recording their own blood pressure and weight in addition to having an individual physical assessment with their provider in the group space [7]. The women then participate in a facilitated group discussion about pregnancy, childbirth, and parenting topics [7]. While each session has an overarching plan and relevant content is covered, the session is led in a facilitative manner which allows the group to contribute to the content [7]. Women also have an opportunity to socialize with each other during the sessions [7].

To date, two randomized controlled trials of group prenatal care have been conducted. One randomized controlled trial, among mostly African American women in the United States, suggested that women who participate in CenteringPregnancy may have a decreased risk of preterm birth, improved prenatal knowledge, greater satisfaction with care, and greater readiness for both delivery and baby care compared to women who receive standard individual prenatal care [8]. Another randomized controlled trial, among American military families, found that women in group prenatal care were more likely to have an adequate number of prenatal visits during their pregnancy and were more satisfied with their care, compared to standard individual prenatal care [9]. However, this study did not find any differences in stress, social support or depressive symptoms [9]. Other studies of group prenatal care have had equivocal findings, potentially as a consequence of research design and measurement, small sample sizes, or different populations [10-15].

The objective of the current study was to understand the central meaning or the core of the experience of group prenatal care for both the women and care providers who participated in CenteringPregnancy through a community maternity clinic. The following research questions guided the study:

• What is the experience of group prenatal care?

• What are women's and care providers' responses to group prenatal care?

This paper focuses on the experience from the perspective of women who took part in group prenatal care.

Although other qualitative studies have described the experience of group prenatal care [16,17], this is the first which sought to understand the central meaning or core of the experience. As such, this study will make a unique contribution by discovering what the experience of group prenatal care means to women. It will provide a depth of understanding of the phenomenon of group prenatal care, going beyond understanding what the experience is *like *for women and seeking to understand the *meaning *of the experience. Providers of group prenatal care can use this information to explain the care model to prospective patients and can also use this information to approach their role with a greater awareness of how group prenatal care impacts women. Given growing interest in group prenatal care, providers and health care systems considering adopting or supporting this model of care can use these findings to inform their decisions and planning.

## Methods

### Design

This study used the qualitative tradition of phenomenology and Heidegger's approach to inquiry to study the experience of group prenatal care [18]. The basic premise was that cultural groups with common experience have common meanings that can provide insight into the experience using the language of the women to find the message of the meaning [18]. Although we suspected that social support would arise as the core phenomenon of interest, we did not review the social support literature before beginning the study. We used bracketing of the theories that we were aware of during the analysis phase of the study to reduce the risk of being influenced by the literature and its projections about what might arise from the data.

### Model of care

Group prenatal care was the model of care being studied, and CenteringPregnancy was the specific model implemented. While many CenteringPregnancy programs include a midwife as the prenatal care provider, this particular CenteringPregnancy program included family physicians as the prenatal care providers. These family physicians were part of a clinic that provides maternity care exclusively to women with low risk pregnancies in Calgary, Alberta, Canada. A physician co-facilitated the group sessions with a perinatal educator. The physicians and educators involved in this group prenatal care program received two days of training in the CenteringPregnancy model through the Centering Healthcare Institute.

As is routine prenatal practice in Alberta, women generally received early prenatal care from their own family physician or a walk-in clinic physician and were referred to the maternity practice for later care. Women who had been referred to the maternity practice or were on its waiting list were contacted and offered the option of participating in group prenatal care. Women were informed that they could attend group prenatal care and discontinue it if they later decided that they preferred to have one-on-one visits. Group prenatal care began between approximately 16 and 20 weeks gestation, and women did not have to pay to attend group prenatal care sessions.

### Recruitment and data collection

Women were recruited to participate in this qualitative study from a cohort study of women who attended the CenteringPregnancy group prenatal care program specified above. For one-on-one interviews, a purposive sample of 8 to 12 postpartum women was planned to facilitate diversity of opinions from women of different cultures and child bearing experience. We planned to complete recruitment when no new data emerged during the interviews.

In May 2009, November 2009, and December 2009, the study team identified women who had recently delivered infants. A research assistant contacted these women by phone and invited them to participate in a one-on-one interview about their experience with group prenatal care. In June 2009, December 2009, and January 2010, one of two interviewers (DAM or MV, both not members of the health care team) met with participants in their homes. The interviews were audio recorded and ranged in duration from about 10 to 30 minutes. Each study participant was assigned a study number, and when the interviews were transcribed verbatim, all names were removed from the transcripts. The central interview question was "What was it like for you to participate in this type of care?" Additional questions included: "What was the best part? What was the worst part? How was this care experience different from what you expected?" The interviewers used probes such as "Can you tell me more about what that was like for you or meant to you?" to assist in further expanding a participant's explanation to gain an understanding of the meaning to the participant.

All participants provided written informed consent, and all names used in this paper are pseudonyms. The study was approved by the University of Calgary Conjoint Health Research Ethics Board.

### Analysis

Before beginning data analysis, each investigator wrote a description of their personal experience of group prenatal care and/or identified their own personal beliefs or theoretical understandings of social support that might influence the way they would be examining the data to address reflexivity [19]. Each investigator read the transcripts independently multiple times to facilitate dependability [19]. To begin the coding process, investigators highlighted and noted in margins statements (issues, highlights, concerns, accomplishments) that had meaning in relation to the group prenatal care experience. Each investigator grouped these statements into "meaning units" or themes and composed a description. The investigative team then met to look for all possible meanings and divergent perspectives (commonalities and unique aspects; the range of responses) and to write a description of the group prenatal care experience. The investigative team met on a regular basis to reach consensus on meaning themes and develop an overall description of the "essence" or core of the experience, returning to the text and their associated meaning statements to ensure dependability [19].

In July 2010, a validation session was arranged to address confirmability [19]. A research assistant invited women to participate in a two-hour focus group of 6 to 10 women and asked each woman to indicate her availability for the two proposed times. A research assistant later confirmed the date of the event and reminded each woman of the event by phone or email. The focus group was held in a meeting room at the research office. At the focus group, DAM shared preliminary findings with study participants and asked women to share their thoughts on the analysis and how it aligned with their own personal experience. The validation session lasted approximately 90 minutes and was audio recorded and later transcribed. Further data collection and validation sessions were planned if needed based on results of the first validation session.

## Results

From September 2008 to July 2010, 77 women had participated in the cohort study on group prenatal care and were eligible to participate in this qualitative sub-study. A total of twelve women (50% of those invited) participated in a one-on-one interview and/or the validation session. Table 1 details the number of women at each stage of recruitment. Of the two women who were invited to participate in a one-on-one interview but declined, one cited being too busy with her infant and the other asked for a call back but a research assistant was unable to reach her. Of the five women who were invited to participate in the validation session but declined, three cited that they would be out of town during the proposed times for the session and two were not interested. Of the eight women who agreed to participate in the validation session but did not attend, one woman called the day of the validation session to inform the research assistant that she could not attend due to a sick infant and the reasons for the other seven women are unknown. Table 2 reports the characteristics of those who participated. The one-on-one interviews occurred approximately 8 to 14 weeks postpartum while the validation session included women who were between 7 and 43 weeks postpartum.

**Table 1:** Number of women in each stage of recruitment

	One-on-One Interview n	Validation Session n	Total N
Available to recruit	40	77	77
Attempted to contact	14	23	33^a^
Invited to participate	10	18	24^b^
Agreed to participate	8	13	18^c^
Participated	8^d^	5	12^e^

^a^There were attempts to contact four women for both an interview and the validation session.

^b^Four women were invited to participate in both an interview and the validation session.

^c^Three women agreed to participate in both an interview and the validation session.

^d^One woman's husband also participated in the one-on-one interview (not included in count).

^e^One woman participated in both an interview and the validation session.

**Table 2:** Characteristics of women who participated in the interviews and validation session

Characteristic	Overall **N = 12**^**a**^ n (%)
Age at time of participation (min = 27, max = 39)	
25 to 29 years 30 to 34 years 35 to 39 years	5 (41.7) 5 (41.7) 2 (16.7)
First time mothers	10 (83.3)
Married/common-law	11 (100.0)
Annual household income, before taxes	
Less than $40,000 $40,000-69,999 $70,000-99,999 $100,000 or more	2 (18.2) 2 (18.2) 3 (27.3) 4 (36.4)
Highest level of education	
Graduated high school Some college, trade, university Graduated college, trade, university Some graduate school	4 (36.4) 3 (27.3) 3 (27.3) 1 (9.1)
Born outside of Canada	5 (45.5)
Non-Caucasian	4 (36.4)

^a^Denominator varies due to missing data for some questions.

**Figure 1:**
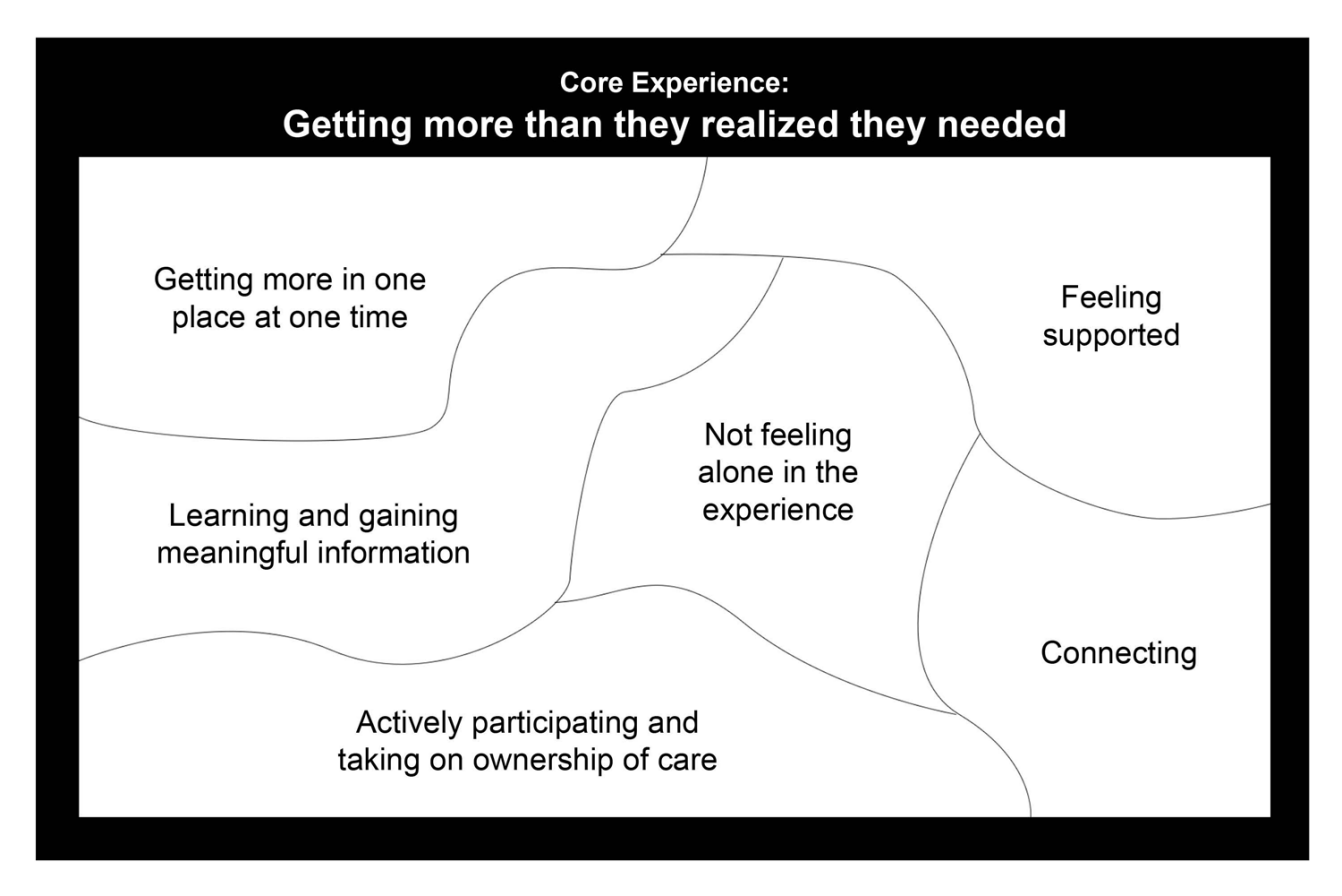
**Women's experience of CenteringPregnancy**.

During the analysis, six themes emerged that described the meaning of the experience to the women participating in group prenatal care and contributed to our perspective of the women's core experience (Figure 1). These themes were "getting more in one place at one time", "feeling supported", "learning and gaining meaningful information", "not feeling alone in the experience", "connecting", and "actively participating and taking on ownership of care". The validation session confirmed these themes and the core experience, which was "getting more than they realized they needed". The themes and core experience are presented in the following sections using exemplars from the participants that best illuminated the themes or statements that summarized the exemplars.

### Theme: Getting more in one place at one time

Group sessions were more efficient than one-on-one visits because women did not spend time waiting for their session to start. They knew the specific time that all sessions would start and when they would end. Women spent more time with their physician and had more opportunity to ask questions. *"Usually at the doctor's office you have to wait... like an hour... so this is only two hours and you're done... it's not so much more time that I'm spending, but I'm gaining more than just a doctor's visit," *(Lisa, 31-year-old first-time mother). Two women in the validation session thought the first few group sessions involved waiting time but indicated, "*It got better... as time went on. And maybe because we knew each other better too, so we would talk a little bit more," *(Nora, 29-year-old first-time mother).

Women thought they learned more than they would have at a standard prenatal visit with just a physician because they received information from the physician, educator, and the other women in the group. "*You can learn from other people... so it's good. It helped me*," (Nicole, 34-year-old, first-time mother). When other women in the group asked questions, women learned things beyond that which they had thought, remembered, or felt comfortable to ask about on their own. Natasha, a 27-year-old first-time mother, stated, *"If I were to just go to the doctor, I wouldn't think to ask about something that hasn't happened." *As time went on, women became more comfortable in asking their questions too because *"you get people who aren't afraid to ask questions, so it makes you feel more comfortable," *(Natasha, 27-year-old first-time mother). On the whole, women felt they got the information they needed and even more information than they realized they needed.

### Theme: Feeling supported

Women felt supported in numerous ways. The physicians paid attention to their concerns and provided women with more than one potential solution or option. At the same time, the group setting and atmosphere helped women realize it was a safe place to ask questions and share. *"So I feel supported, I feel that when I have questions I can... have them answered," *(Lisa, 31-year-old first-time mother). Furthermore, the physicians, the educators, and in particular the other women were a source of rich information and knowledge that one *"couldn't get from books," *(husband of Rebecca, 30-year-old first-time mother). As one woman said, *"It's nice that somebody can... give you advice and you can give them advice," *(Natasha, 27-year-old first-time mother).

Women in the group also supported each other by listening to each other and giving each other time to share. Women were *"... not fighting over people to get [their] words in and... everybody had their time," *(Natasha, 27-year-old first-time mother). Another woman said, "*It was nice and good to know everybody... and share the experience with them*," (Priya, 27-year-old mother with other children). Through this, women received emotional support from the other women in the group. "*They understood where I was, I understood where they were and... we all just became friends," *(Rebecca, 30-year-old first-time mother). Women could share their experiences and feelings with other women in the group, including things they thought their friends outside of the group might not want to talk about. One woman who had been through multiple pregnancies reported, "*I tell everyone I've never had this much support and it really helped... it's the first time I didn't get postpartum depression and I think having all this support during my pregnancy is what kind of eliminated that*," (Robyn, 38-year-old mother with other children).

### Theme: Learning and gaining meaningful information

Women reported that the information they gained in group prenatal care was useful, practical, detailed, up to date, and tailored to their needs. *"I liked that each day was... a different topic, and... the [educator] would always ask if there was anything you wanted to discuss next time... that way everybody got what they needed," *(Rebecca, 30-year-old first-time mother). The information was especially helpful for first-time mothers. *"I think the information that I received was very valuable... very helpful detailed information, especially preparing for the labour part... I didn't know what to expect, so it was really helpful to be able to get information about those things," *(Lisa, 31-year-old first-time mother). As women's relationships developed over time within the group, the information became more meaningful. Feeling a sense of connection and support in the group, women interacted with others in a way that shared some of their deepest needs, fears, questions, concerns, and experiences.

When women's partners attended group sessions with them, women reported increased involvement from their partner and partners "*got to know... about all our aches and pains*," (Robyn, 38-year-old mother with other children). In addition, women felt supported and secure when their partners attended. *"My husband learned a lot too... and he helped me with everything... at the end, like at delivery time, he was with me. So... actually I felt more safe," *(Alia, 27-year-old first-time mother).

### Theme: Not feeling alone in the experience

Talking to *"other women going through the exact same thing at the exact same time as you," *(Natasha, 27-year-old first-time mother) helped women to "*feel normal... like I'm not the only one," *(Naomi, 31-year-old first-time mother). Even women who had gone through pregnancy before felt this way. "*I wish... this group existed when I had my first, 'cause... I felt really alone with my first.... And this time... to be in that group... it's like, 'Wow, other people do feel like that*,'" (Robyn, 38-year-old mother with other children).

Women identified with each other and understood where other women were at. For some women this was more than they knew they needed. One woman stated, *"At first [the sessions] were... I didn't find them useful. I thought they were quite... touchy feely, not very concrete... we talked about how we felt," *(Lisa, 31-year-old first-time mother). Yet later in the interview she acknowledged, *"It helped a lot to talk to people and... oh you have this happen too? Or you feel this way too? So... to identify with... the people there. It was very good for me."*

### Theme: Connecting

Women saw one of two physicians over the duration of their prenatal care. As a result of this consistency, the additional time, and the type of encounter, women said they developed a connection with the physicians and "*buil[t] a relationship with them which was nice," *(Angela, 39-year-old first-time mother). This relationship increased women's level of comfort with the physicians, which enabled women to feel confident in asking their questions and sharing their thoughts. *"I had more of a connection I feel with Doctor S because I saw her more. But I mean, just the connection that I felt with her I felt so comfortable asking questions," *(Rebecca, 30-year-old first-time mother). Women thought the physicians were more aware of what care had been provided to date, and women did not have to explain themselves and their history at each visit.

Women experienced a connection with other women in the group in going through the pregnancy experience together and sharing along the way. This was particularly meaningful for those who did not have friends who were pregnant at the same time. *"Lots of my friends haven't had kids, so they don't understand exactly... where somebody who's going through the same thing will talk to you about it," *(Natasha, 27-year-old first-time mother). The universal pregnancy experience and sharing about their lives allowed connections to develop and facilitated the development of friendships for some women. Some of these friendships endured beyond the group.

### Theme: Actively participating and taking on ownership of care

Women actively participated in the care of themselves and their babies by eliciting the information that was important to them. Women learned what they could expect and came to understand what they were experiencing or might experience next. *"And they prepare you for it, whether it be scary or not but at least you know what to expect," *(Natasha, 27-year-old first-time mother).

Women became knowledgeable, had a greater awareness of their own health, and learned to care for themselves and the baby growing inside of them. Women learned what to worry about and what not worry about and knew what to do in previously unknown circumstances. *"...'Cause my water breaks and I knew that... I'm supposed to go to the hospital...[at the hospital] they gave me two options... you can go home [and] come back, or... you can stay here. Because... they taught me... when your water breaks, your baby's at risk. I'm like, '... I'll just stay here,'" *(Alia, 27-year-old first-time mother).Knowledge became power for women because as Natasha, a 27-year-old first-time mother, said *"The more you know, the better you can handle a situation."* Women became more responsible for their own health and were more involved in their care than at a regular physician appointment.

*"You take your own blood pressure. You check your own urine. So I think it also helped... us to... take ownership of our own care... you're doing something for yourself to take care... so not just to go sit in a doctor's office and be told to... pee in a cup. And they don't even tell you what it means or what they check," *(Lisa, 31-year-old first-time mother). However, this level of responsibility created some discomfort at times for one mother who found that "*occasionally I was worried about the accuracy of my own [blood pressure] readings*," (Nora, 29-year-old first-time mother).

### The core of the experience: Getting more than they realized they needed

Although women both contributed to and received from the group, women focused on what they gained from the group (physicians, educators and other women) rather than what they gave when describing their experience. The group allowed women to share the pregnancy experience together and information with each other while also allowing physicians and educators to contribute their expertise. Through this kind of sharing, women found more in one place at one time, learned and gained meaningful information, connected with others, did not feel alone in the pregnancy experience, felt supported, and actively participated in their care. As women went through the pregnancy experience and prepared for parenting a newborn, *"it was nice that... everything I needed was right there," *(Natasha, 27-year-old first-time mother).

Encountering similar needs and experiences in the group, women did not feel alone in their experiences but rather felt supported and connected with other women. At the same time, the diversity of needs, experiences and ideas in the group resulted in women gaining more in one place at one time. As the group had its needs met, each woman also had her individual needs met. *"So there is actually [a] very good balance of both... practical information and also the emotional support... so I think anyone could benefit [from] at least... some part of it," *(Lisa, 31-year-old first-time mother).

When women came to the group, some had ideas of what they needed, others realized they had needs but were not entirely clear on what those were, while others were unaware of some of their needs. Regardless of the level of awareness that women had of their needs, they came away with more than they realized they needed, whether it was information, feeling normal in identifying with other pregnant women, or something else. Women expected to get some medical care but ended up getting so much more than that. *"I just want to say thank you because I never expect... to have... this care when I go to my family clinic," *(Nicole, 34-year-old, first-time mother).

## Discussion

The central meaning of the experience of group prenatal care for women in this study was getting more than they realized they needed. Although these results do not indicate that women are not getting what they need from individual care, the results suggest that women can gain more from group prenatal care than from individual care. In individual care, the physician has very limited time, and the woman tries to get what she thinks she needs in that time. The brief prenatal visit does not allow much time to develop a connection with the physician, women may not be as comfortable asking questions, and women do not actively participate in their care. Consequently, the information provided by the physician may not meet the underlying need or question that the woman has. Moreover, women do not have the same opportunity to learn from others in an individual visit as there is no one else to ask the questions that she has not thought to ask, is not comfortable asking, or has forgotten to ask. In individual care, women also do not have the same sense of ownership with regards to their health and prenatal care.

It is also important to note that while women may have received more than they realized they needed through group prenatal care, each woman may have experienced this core in a slightly different way based on their experience of the themes within the core. Some women may have found that they received more than they realized they needed by learning that they were not alone in the experience of pregnancy and by actively participating in their care. Others may have received more than they realized they needed because they learned and gained a vast amount of meaningful information. Regardless of the variation in experience of the themes, group prenatal care provided more than women realized they needed.

While the current study used the phenomenological method to *understand the central meaning or core of *the experience of CenteringPregnancy, other studies have used other qualitative methods to *describe *the experience of CenteringPregnancy. A study by Teate et al. among Australian women found that CenteringPregnancy used women's time well, offered a supportive environment, improved learning, provided a sense of being normal, and provided opportunities to develop relationships [20]. These themes paralleled the following themes in the current study: "getting more in one place at one time", "feeling supported", "learning and gaining meaningful information", "not feeling alone in the experience", and "connecting". Other qualitative studies have been conducted among specific sub-populations. Kennedy et al. conducted a study among women in an American military setting and found that women described the experience as "I wasn't alone" [16]. Their experience included an aspect of normalization and identification with other pregnant women as well as a sense of community and friendship, closely aligning with two of the six themes in the current study - "not feeling alone in the experience of pregnancy" and "connecting". Another study by Novick et al. among African American and Hispanic women in the United States described six themes [17]. While the themes identified by Novick et al. had some alignment with those identified in the current study, there was particularly clear alignment regarding "learning and gaining meaningful information" [17]. To summarize, the themes found in the current study align well with previous research. This provides additional confidence in the validity of the themes identified.

Overall, there were strong elements of social support reflected in the women's experiences in this study. Most commonly, social support has been conceptualized (and measured) by looking at different forms of social support [21-23]. The basic assumption among these concepts is that there are different forms of social support that meet distinctly different needs--from material or tangible needs, to needs for information or advice, to needs for encouragement, affection, identification, or a sense of belonging. Indeed, women in group prenatal care had a variety of needs met, such as being connected to other women in the group (positive social interaction), not feeling alone in the experience of pregnancy (emotional support), and learning and gaining meaningful information (informational support). Furthermore, a number of social support theories note that support must be perceived as such by the recipient [23-26], and this was found to be true with women feeling supported in group prenatal care.

In addition, the findings of this study advance our understanding of social support beyond current conceptualizations. Some have suggested that needs must be identified before support can be given, but women in group prenatal care found support for needs they were aware of and for needs they did not realize they had [24-27]. Moreover, the theory of mutual intentionality posits that the giver and recipient of social support make active decisions to give and receive support [25,26]. While it was clear that women in group prenatal care supported each other, it seemed more so that women gave support to others as they sought to have their own needs met in the group and less so through an active, conscious choice. Indeed, the experience of social support in a group context may vary when compared to social support offered by one individual to another.

Finding elements of social support in women's experience of group prenatal care was not surprising as assessment, education and support are core program components of CenteringPregnancy [28]. The structural elements of the CenteringPregnancy groups have been designed to facilitate the development of social support [7,28]. Each session takes place in a circle and is facilitated rather than directed; women's contributions are valued, and there is time to talk socially with other women [7,28]. The shared experience of pregnancy is also thought to promote relationships among women in the group [28].

Social support has been theorized to impact health, either through a direct effect or a buffering effect. It would be plausible then that group prenatal care, through providing an experience like that of social support, would also lead to improved health outcomes. Programs specifically designed to provide social support have often focused on the instrumental or tangible needs of pregnant women like child care or transportation [29]. While these tangible needs are important, group prenatal care may additionally meet the intangible needs of women. Women actively participate in their care and have more meaningful information to use as they progress in pregnancy, deliver their infant, and ultimately become parents. Women may have lower stress levels as they feel connected with others, supported, and empowered. Lowering stress levels may affect the physiology of the pregnant woman and her fetus and is a potential pathway through which group prenatal care may lead to improved birth outcomes such as lower rates of preterm birth, as found in Ickovics et al. [8].

As this study indicates, group prenatal care has the potential to enhance the care experience for women in community prenatal care clinics. Group prenatal care may also have the potential to change the experience of social support for marginalized groups, such as pregnant adolescents, who describe having to "piece together" social support from available sources [30]. In group prenatal care, women are connected through their pregnancy experience and have access to more than they realize they need in one place at one time, regardless of parity, ethnicity, economic means, and age.

### Limitations and other considerations

The women who participated in the interviews were those who chose to complete the program or stay with the program as far as they were able (e.g. one woman was ordered on bed rest and could no longer attend group prenatal care). We do not have perspectives from those women who dropped out of the program, and their experiences are likely to have differed from those who stayed. Also, the sample for this study largely consisted of first-time mothers. While this could mark a limitation to the current study, the data gathered from the two mothers with other children suggest their experience of CenteringPregnancy was consistent with that of the first-time mothers.

The two participants who thought a group prenatal care session may not be as efficient as a physician's visit attributed this to the time spent waiting in the first few group sessions. Both reported less waiting in later sessions and seemed to suggest that some amount of "sitting around and waiting" in the first few sessions may be necessary for women to become comfortable enough to interact with each other during the session. Also, some providers may have been more skilled at facilitating interaction among the women in earlier sessions. However, for those not planning to take prenatal classes, group prenatal care may be perceived as less efficient.

Perceived inefficiency may have acted as a barrier to recruitment and retention for some women. Another aspect of the program that may have been a barrier for some women was discomfort with the level of responsibility required for self-care assessments (e.g. blood pressure measurements). However, we did not conduct an analysis of factors that acted as barriers and facilitators to participation in the group prenatal care program. Our focus in this paper was on group prenatal care as a phenomenon and not on the decision to participate in group prenatal care.

Although women reported that group prenatal care gave them more than they realized they needed, some expressed a desire for more postpartum and parenting information as also found in another study [20]. This may indicate a gap in information and support after delivery and the opportunity to continue to support women in the postpartum and parenting stages.

## Conclusions

While some have suggested that cultural values may lead to different social views on the need for social support [26,31], many cultures have embedded practices where people interact in groups and these have endured over time [32]. For instance, circles of women are an ancient tradition where women share values and beliefs and where women learn from one another [33]. Yet even in a contemporary North American society where independence is valued, the results of this study suggest that group prenatal care could play a key role in addressing women's needs and improving health outcomes through the influence of information and social support.

## Competing interests

The authors declare that they have no competing interests.

## Authors' contributions

DAM and SCT conceived and designed the study. DAM and MV acquired the data. All authors contributed to the analysis and interpretation of data. DAM and MV drafted the manuscript, and SMD, JS, SH, and SCT revised it critically for important intellectual content. All authors read and approved the final manuscript.

## Authors' information

DAM is the director of the department of Public Health Innovation and Decision Support within Alberta Health Services and an adjunct associate professor in the Faculty of Nursing at the University of Calgary. MV is a project coordinator with the Department of Paediatrics at the University of Calgary and a research assistant with the Public Health Innovation and Decision Support department at Alberta Health Services. SMD is an associate professor of Clinical Obstetrics & Gynecology and Women's Health at Albert Einstein College of Medicine/Montefiore Medical Center. JS is a senior analyst in the department of Public Health Innovation and Decision Support within Alberta Health Services. SH was a research assistant with the Department of Paediatrics at the University of Calgary and the Public Health Innovation and Decision Support department at Alberta Health Services. SCT is a professor in the departments of Paediatrics and Community Health Sciences at the University of Calgary.

## Pre-publication history

The pre-publication history for this paper can be accessed here:

http://www.biomedcentral.com/1471-2393/12/17/prepub
